# Participatory design of built environment strategies to enhance movement, wellbeing, and quality of life among incarcerated men

**DOI:** 10.1186/s12966-026-01871-7

**Published:** 2026-01-22

**Authors:** M. Giné-Garriga, C. Palma, S. Signo, C. Isanta, R. Romeva, S. A. Portillo, A. C. King, M. A. Cortés, D. Ballester, E. M. Sebastiani

**Affiliations:** 1 School of Psychology, Education and Sport Sciences Blanquerna, Universitat Ramon Llull, Barcelona, Spain; 2 School of Health Sciences Blanquerna, Universitat Ramon Llull, C/Padilla 326, 08025 Barcelona, Spain; 3 Department of Epidemiology and Population Health, Stanford University School of Medicine, Palo Alto, CA USA; 4 Department of Justice and Democratic Quality, Government of Catalonia, Barcelona, Spain; 5 Sport Department, Municipality of Badalona, Barcelona, Spain

**Keywords:** Prison setting, Physical activity, Sedentary behaviour, Co-creation, Community science, Our Voice method

## Abstract

**Background:**

The global prison population has grown by 5% since 2020, intensifying existing challenges to inmate health and well-being. Incarcerated individuals experience disproportionately high levels of sedentary behaviour (SB), which increases their risk of physical and mental health problems. Although regular physical activity (PA) can mitigate these risks, correctional settings often present environmental and institutional barriers that limit opportunities for movement. While modifying built environments has shown promise for promoting PA in community settings, limited research has explored these strategies in prisons. This study applied a community science, co-creation approach to identify inmate-informed, context-specific solutions to reduce SB and enhance PA within a prison setting.

**Methods:**

The study was conducted in a Spanish male prison with 26 adult inmates. Based on facility-use records and self-reported activity/sedentary levels, 13 highly active and 13 inactive inmates were purposively selected to capture diverse perspectives. Participants took part in structured workshops using the Our Voice community science framework. With the Our Voice Discovery Tool mobile app, they documented environmental barriers and facilitators to PA through geo-tagged photos and audio or text comments while moving through prison spaces. Data sources included workshop transcripts, facilitator notes, and app-generated digital content, which were analyzed using Braun and Clarke’s codebook thematic approach. Participants then proposed and ranked potential solutions using an Eisenhower Matrix, and these were later refined with input from prison staff and policymakers.

**Results:**

Six major themes influencing movement opportunities emerged: (1) activities, (2) spaces, (3) equipment and materials, (4) staff and support, (5) regulation-related limitations, and (6) scheduling. In total, 39 inmate-generated strategies were identified, 31 of which were rated as high priority. Low-difficulty actions included improved maintenance of activity areas, expanded access to equipment, and more flexible use of existing spaces. Participants emphasized that socially supportive environments were essential to motivation, adherence, and mental well-being.

**Conclusions:**

The study demonstrates the feasibility and value of participatory community science in correctional settings. Involving incarcerated individuals in designing PA solutions generates practical, context-appropriate strategies and supports greater health equity. This approach offers a scalable model for institutions aiming to reduce SB and promote PA through built-environment improvements.

## Background

The global prison population has increased by about 5% since 2020, despite efforts to reduce incarceration in many countries [1]. Incarcerated people face substantial health challenges: imprisonment is associated with physical inactivity, smoking, substance use, poor diet, and other behaviors that elevate the risk of chronic and acute physical and mental health conditions [2–4]. Sedentary behavior is particularly prevalent; for example, male and female incarcerated adults average fewer than 7,100 and 6,500 daily steps, respectively [5], and recent research shows that most of their day is spent sedentary with limited light or moderate activity [6].

These health risks impose major economic burdens. Elevated healthcare needs during incarceration, high rates of hospitalization after release, and reduced employability generate substantial costs for communities and health systems. In the U.S., prison healthcare and downstream societal costs amount to tens of billions of dollars annually [7]. From a public health perspective, achieving health equity, defined as the condition in which “everyone can attain their full potential for health and well-being” [8, 9], requires attention to populations often excluded from mainstream health promotion. Incarcerated persons represent exactly such a group: historically marginalized, rarely consulted in policy or program design, and disproportionately burdened by illness. To reduce unjust health disparities, it is essential not only to measure health behaviors in prisons, but also to engage incarcerated individuals in research and decision-making that shape the environments in which they live [10].

Physical activity (PA) and reduced sedentary behavior (SB) are well-established contributors to physical and mental well-being in the general population [11–15]. Yet among incarcerated populations, evidence remains limited and often focuses on structured exercise programs rather than everyday movement under carceral conditions. Co-designed interventions, such as Fit for LIFE, show promise in increasing PA and reducing SB but remain rare [16].

The physical (built) environment within prisons is a crucial but underexplored influence on PA and SB. While social factors (such as peer relationships, institutional rules, and supervision) are known to shape behavior, the design and layout of correctional facilities (e.g., including cell size, common areas, access to outdoor or recreational space, lighting, and safety) may strongly constrain or enable movement [17, 18]. For example, architectural features such as poor lighting, limited outdoor contact, and constrained circulation spaces have been linked to stress, low PA, and passive leisure activity among incarcerated people [19].

To address these gaps, community science (or citizen science) offers a powerful methodological approach to address these gaps. It enables people with lived experience to participate in data collection, interpretation, and action [20, 21]. In correctional settings, peer-led and community-engaged models can enhance trust, relevance, and feasibility of research [22]. The Our Voice method combines mobile-enabled data gathering with participatory action and has successfully engaged underserved groups in evaluating and improving built environments [21, 23]. However, to date, this method has not been applied in prison settings. This gap represents both a major challenge and a unique opportunity to bring incarcerated individuals into co-creation of health-promoting built environments.

Environment-driven, participatory strategies to promote PA in prisons may reduce aggression, improve social skills, support rehabilitative climates, and enhance post-release reintegration [24]. Yet PA promotion efforts in countries such as Spain, Greece, and the United Kingdom have often been limited to small amounts of scheduled activity (e.g., 90 min per week) and cannot meet most inmates’ needs or motivations [25]. Evidence-based, participatory approaches that elevate the voices of incarcerated people are urgently needed to design feasible and acceptable solutions.

Given the large number of individuals who cycle through prisons each year, improving inmate health has important implications for community well-being, public safety, and health equity [4]. This study aims to identify features of the prison-built environment that can promote movement—increasing PA and reducing SB—through a co-creation process using the Our Voice citizen-science method.

## Methods

This study followed the Consolidated Criteria for Reporting Qualitative Research (COREQ) guidelines for qualitative interviews and focus groups [26].

### Research team and reflexivity

The workshops and data collection activities were facilitated by a team of six researchers from a local university. The team included both male and female academics with advanced training in PA, public health, psychology, and qualitative research methods (PhD and senior researchers). Most facilitators had prior experience conducting qualitative studies, leading co-creation processes, and/or applying community science approaches. Researchers had no prior personal or professional relationships with incarcerated participants. Before participation, inmates were informed that the researchers were academics interested in understanding environmental influences on PA and SB and in identifying strategies to improve movement opportunities and well-being in the prison setting collaboratively. Researchers emphasized their neutral role, academic motivations, and commitment to fostering a safe, respectful environment for open discussion. Reflexive practices, including memos and team discussions, were used throughout data collection and analysis to monitor assumptions, document decision-making, and ensure interpretations were grounded in participant perspectives.

### Study design and methodological orientation

We employed a qualitative, inductive design integrating three components: (1) the Our Voice community science method, (2) co-creation workshops, and (3) Braun and Clarke’s codebook thematic analysis. This approach enabled exploration of how the prison environment influences PA and SB and facilitated collaborative development of feasible strategies for improvement with both incarcerated residents, staff and a policymaker.

### Participant selection and recruitment

Recruitment targeted adult male residents from four housing units in a single prison selected for its high prevalence of mental health concerns, frequent violent incidents, and administrative support for implementing well-being initiatives. Information about the study was provided during regularly scheduled group sessions that residents already attended, facilitated by different professional staff members. All residents who expressed interest were invited to a prescreening phase in which prison records on sports-facility use, self-reported PA and daily sedentary time (assessed with the International Physical Activity Questionnaire), and demographic information were reviewed.

To capture contrasting perspectives on activity experiences and environmental needs, purposive sampling was used to select participants from both ends of the activity spectrum. This included 13 residents with the highest levels of PA and 13 with no documented use of sports facilities, supported by low self-reported PA and more than nine hours of sedentary time per day. A total sample of 26 was chosen to ensure manageable group sizes for co-creation workshops and community-science activities, facilitating meaningful engagement and safe implementation within the prison environment. No participants withdrew after enrollment. The final sample represented variation in age, sentence length, housing unit, and levels of activity and sedentary behavior.

### Staff participants

Staff members were purposively selected based on their decision-making authority and operational roles related to the prison environment and movement opportunities. The staff sample included four members of the prison administration and 1 external policymaker. Participants included the prison director, head of sport technicians, social educator, head of security, and one policymaker from the Department of Justice. Their roles allowed them to influence policy, oversee recreational spaces, manage daily routines, and ensure safety, making their perspectives essential for co-creating feasible interventions. Staff were invited through institutional communication channels and in-person meetings coordinated by prison administration.

### Setting

At the time of recruitment, the prison housed 1,257 men across seven housing units. In consultation with the prison director, four units were selected for inclusion in the study. Each unit contained an outdoor communal area estimated at approximately 650 m^2^; these dimensions were based on staff approximations rather than precise measurement. Units also included an indoor shared space furnished with tables and chairs, along with several classrooms used for organized activities led by different professionals. Outdoor areas typically offered some gym-type equipment, such as free weights, balls, and rackets, although the quantity and condition varied across units and no standardized distribution system was in place. Residents spent most daytime hours in these shared spaces, as access to their cells was limited to a two-hour period after lunch and then again at bedtime following the evening meal. In addition, each resident was permitted to use the central sports facility once per week for a 90-min session, which provided access to an indoor gym with basic equipment and an indoor multipurpose court.

All data collection occurred inside the prison. Workshops took place in designated meeting rooms provided by the administration. The community science activity was conducted during supervised walks through shared prison spaces, accompanied by a staff member or researcher. Only participants and researchers were present in the workshop rooms; however, prison security personnel were occasionally visible in surrounding areas during the observational walks as required by institutional protocols.

### Co-creation process and community science

The co-creation process involved three sequential 2-h workshops with each inmate group (n = 13 per group). All workshops followed a structured facilitation guide that outlined objectives, step-by-step activities, discussion prompts, and follow-up probes to ensure consistency across sessions and facilitators.

#### Workshop 1: Community science data collection

In the first workshop, participants became community scientists using the Our Voice Discovery Tool mobile application on TracFone devices. The Our Voice Discovery Tool is a mixed-methods mobile app that lets users document features of their environment that help or hinder PA. Users take photos, record or type short narratives, and provide simple emoji-based ratings. The app also creates a walking map of their route. All data are anonymized. The tool is easy to use and has been successfully used by people of all ages, including adults over 95 years old [27]. Facilitators introduced the method using a brief scripted orientation covering: (1) the purpose of community science, (2) how to capture photos and narrations, and (3) ethical guidance on photographing only spaces, not people.

Participants then worked in small groups (3–4), each accompanied by a staff member or researcher. To prevent clustering by existing social relationships or activity levels, and to avoid contamination in the picture-taking process by limiting influence among participants who routinely share spaces, participants were randomly allocated to these groups. Groups conducted a 2-h observational walk, capturing photographs and audio or text reflections in response to the standardized prompt: “What spaces within your environment facilitate or hinder movement or sport practice during the day?”.

In this European prison context, sport refers broadly to PA (structured or unstructured). Although SB was not explicitly included in this opening prompt, facilitators used optional probes to elicit early reflections relevant to SB when appropriate, including: “Are there spaces where you feel you spend too much time sitting or waiting?”; “What makes it easy or difficult to move around during the day?”.

These data were automatically geo-tagged, time-stamped, and uploaded into the Discovery Tool system for analysis in Workshop 2.

#### Workshop 2: Review and collective analysis of community science data

Workshop 2 was conducted separately with the two inmate subgroups (most active and least active) and three researchers to review and analyze the Discovery Tool data. All facilitators, staff or researchers, followed a shared protocol with predefined discussion questions and analytic steps, ensuring consistency across groups. The workshop followed three structured stages:

- Review of collected data: participants viewed their geo-tagged photographs and narrations projected on a screen. Facilitators used guiding questions such as “what do you see here?”, “why does this space matter for movement or daily routines?”, “what is happening in this spot during a typical day?”.
- Discussion of PA, SB, and health: facilitators introduced brief prompts linking observations to PA, SB, and emotional/physical well-being. Examples included: “how does this space affect how much you move?”, “are there places where you feel you spend long periods sitting or inactive?”, “how do these spaces influence how you feel during the day?”.
- Identification of barriers, facilitators, and strategy generation: participants collaborated to identify key environmental barriers/facilitators and to propose strategies to increase movement and reduce sedentary time. Probes included: “What would make this area easier to use for activity?”, “what barriers could realistically be changed?”, “what ideas do you have that might work within the prison’s rules and routines?”.

All data were anonymized before analysis, and the research team independently conducted final coding.

#### Workshop 3: Strategy prioritization

The third workshop was also conducted separately with the two inmate subgroups. Using a structured worksheet and an adapted Eisenhower Matrix [28], participants evaluated each strategy generated in Workshop 2 according to importance (impact on movement/SB), and perceived feasibility (within institutional constraints). Facilitators used prompts such as: “how important is this change for improving daily movement?”, “how realistic is it within current rules, staffing, and resources?”. This resulted in a prioritized list of inmate-generated strategies.

#### Staff and policymaker workshops

Two additional 2-h workshops were held with prison staff and a policymaker: Workshop A. Interpretation and system-level reflection: staff reviewed the inmate-generated findings and engaged in structured reflections guided by questions such as: “what operational factors influence these barriers or facilitators?”, “how does the daily schedule affect opportunities for movement?”.

Workshop B. Feasibility review and implementation planning: staff evaluated each inmate-proposed strategy for operational feasibility, safety, and resource requirements. This session served as a collaborative planning forum, resulting in a refined list of viable strategies and outlining implementation steps for those considered most feasible and actionable.

### Data collection

No pilot testing was required due to the validated nature of the protocol. All sessions were audio-recorded, and researchers took field notes during and after each session. The duration of each workshop was approximately two hours. Transcripts were not returned to incarcerated participants for revision due to institutional constraints; however, interim findings were discussed in subsequent sessions, providing opportunities for participant feedback (member checking).

### Data analysis

All workshops were audio-recorded in full and transcribed verbatim. Data analysis followed Braun and Clarke’s codebook thematic analysis, using an inductive and iterative approach [29]. The analytic dataset included: (1) workshop transcripts, (2) facilitator field notes, and (3) multimodal outputs from the Our Voice Discovery Tool (geo-tagged photographs, written descriptors, and audio narratives). These materials were imported into Open Code qualitative software (version 4.03) to organize transcripts, code textual and visual data, and maintain analytic memos.

Given the interconnected nature of the workshops, all data were analyzed as a single integrated dataset. This approach reflects that insights generated in one session were often revisited, refined, or challenged in subsequent workshops. Metadata on group membership and session type (active inmates, inactive inmates, staff workshops, policymaker input) were retained to allow comparisons within and across participant groups and to identify convergence or divergence of perspectives.

Four researchers participated in the analysis. To enhance rigor and reduce individual bias, coders worked in two independent pairs. Each pair conducted an initial round of open coding across the full dataset, including transcript segments, Discovery Tool reflections, and descriptions of photographs, generating preliminary codes grounded in participants’ spoken and visual observations.

The full team then compared coding decisions, resolved discrepancies through discussion, and collaboratively developed a shared codebook. The codebook included code names, definitions, and representative quotations with photographic captures. Reflexive memos documented analytic decisions, emerging insights, and assumptions throughout the process. Regular consensus meetings were held to collapse overlapping codes and compare patterns across participant groups.

Through iterative coding, comparison, and discussion, preliminary codes were consolidated into higher-order themes. Theme development was inductive and data-driven, informed by constant comparison across multimodal Discovery Tool outputs, workshop discussions, and inmate and staff perspectives.

The final thematic structure was reviewed for internal consistency and distinctiveness. Member checking was conducted indirectly during subsequent workshops, where preliminary interpretations were shared and refined based on participant feedback, which is a suitable method in the prison context, where direct transcript review was not feasible. Selected quotations, photographs, and narrative entries were used to illustrate themes and triangulate textual and visual accounts of environmental experiences.

## Results

### Participant characteristics

Twenty-six incarcerated adult males participated in the study (mean age = 39.7 ± 9.4 years). Educational attainment ranged from no formal education (30%) to university-level education (2%), with the majority completing primary (46%) or vocational (16%) training. Four prison staff members (prison director, head of sport technicians, social educator, head of security officers), one policymaker, and six researchers were also involved. Among inmates, 13 were categorized as highly active, regularly attending the prison sport facility for the full 90 min offered each week, and with higher self-report levels of PA and lower daily sitting time of 9 h according to IPAQ.

### Themes and exemplar quotations

Thematic analysis of workshop transcripts, field notes, and outputs from the Our Voice Discovery Tool identified six major themes representing key domains for potential movement-promoting strategies. Table 1 presents each theme with its definition and grounded interpretation, with an accompanying participant quotation for context. Table 2 provides an overview of the themes along with illustrative participant photographs and quotations.

**Table 1:** Themes, definition, and selected quotes

Theme	Definition and grounded interpretation	Quotes*
Activities—Availability and structure of physical activity opportunities	Participants described the types of physical activities available to them, including organized sessions, informal activities, and opportunities for self-directed movement. Descriptions focused on how activities were offered, scheduled, or left unstructured. Across both groups, participants noted that limited structure and inconsistent organization affected their ability to engage in activities. Highly active inmates expressed a desire for more frequent and better-organized sessions, while less active inmates emphasized the absence of guided activities as a barrier to participation	*"I would join a football session every day if someone would organize it properly; right now, it’s just random."* – Participant 7, inactive
Spaces—Characteristics and use of physical spaces	Participants discussed the condition, accessibility, and suitability of indoor and outdoor spaces within their housing units and the prison sports facility Participants frequently described spatial limitations, including overcrowded yards, restricted access, and inadequate designated areas for movement, that influenced their ability to be active. Active inmates reported adapting to available spaces, while inactive inmates more often highlighted environmental barriers as reasons for not engaging in movement	*"The yard is too small and often crowded. I can’t run freely, so I just stay inside."* – Participant 15, active
Materials/Equipment—Access to and condition of equipment and materials	Participants detailed the availability, quality, and distribution of equipment such as balls, weights, and basic fitness materials Both active and inactive inmates noted that missing, damaged, or inconsistently distributed equipment limited opportunities for activity. Active inmates emphasized the need for reliable equipment to support regular exercise, while inactive inmates described missing materials as reducing motivation to participate	*"Some of the equipment is broken or missing. We can’t play basketball with only one ball."* – Participant 23, active
Staff/Support—Staff presence, support, and facilitation	Participants described the role of staff such as sports technicians, officers, and educators in facilitating, supervising, or motivating participation in physical activity Participants consistently highlighted that staff presence influenced activity levels. Active inmates described sessions running more smoothly when staff facilitated them, while inactive inmates reported that the absence of guided or supervised activities contributed to sedentary routines	*"When there’s no instructor, most guys just sit around. Having someone to encourage us would help a lot."* – Participant 13, inactive
Regulations/Limitations—Institutional regulations and restrictions affecting movement	Participants described prison regulations, rules, and security restrictions that shaped access to spaces, equipment, and organized activities Inmates identified institutional procedures such as strict scheduling, limited group mixing, and movement rules as constraints that reduced opportunities for activity. These restrictions were experienced as structural barriers regardless of participants’ activity levels, though active inmates tended to express frustration more frequently	*"Sometimes we want to go to the gym, but rules say only certain groups at certain times… it’s frustrating."* – Participant 20, active
Schedule/Time allocation—Daily schedules and time allocation for activity	Participants discussed how the daily prison schedule, including work hours, mandatory routines, and limited free time, shaped their opportunities to engage in activity Participants reported that tightly structured daily routines left little time for physical activity. Active inmates expressed a desire for additional or extended training slots, while inactive inmates noted that fragmented schedules made it harder to start or maintain activity habits	*"If the schedule allowed more sessions, I could attend three times a week instead of just once."* – Participant 14, active

^*^All quotes translated from Spanish/Catalan

**Table 2:**
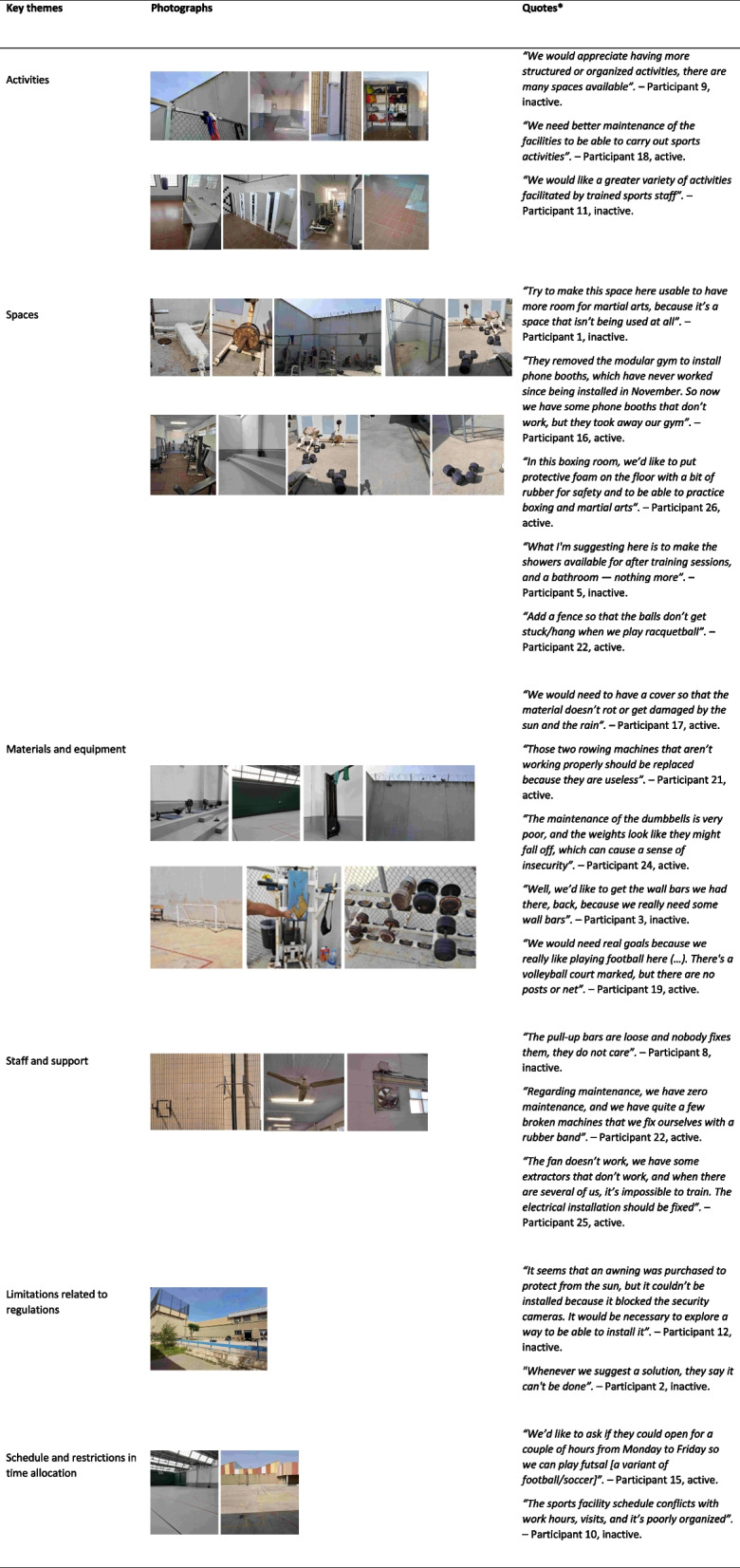
Themes with illustrative photographs and additional quotes from incarcerated participants

*All quotes translated from Spanish/Catalan

Using the Our Voice Discovery Tool, participants captured 46 geo-tagged photographs with accompanying audio or text comments, documenting environmental facilitators and barriers. Photos highlighted limited equipment, and underutilized areas that could be repurposed for PA.

### Strategy identification and prioritization

Across workshops, participants and researchers co-created 39 potential strategies to enhance PA and reduce SB. Strategies were categorized according to the six themes and prioritized using an adapted Eisenhower Matrix based on perceived importance and ease of implementation.

Highly active inmates prioritized 23 strategies, emphasizing equipment upgrades, facility maintenance, and flexible schedules. Less active inmates prioritized 8 strategies, focusing on structured guidance from trained instructors and motivational support. Table 3 shows the prioritized strategies for promoting movement, categorized by participant activity level and implementation difficulty. Group prioritization ensured that strategies from both ends of the activity spectrum were included. Strategies deemed challenging but potentially feasible were flagged for staff review.

**Table 3:** Prioritized strategies for promoting physical activity, categorized by participant activity level and implementation difficulty

Theme	Higher PA levels	Lower PA levels
Activities	Offer more organized activities – increase the number of sports staff	Organize directed sessions (e.g., strength training circuits) to increase motivation and engagement in physical activity Allow for external sports teams (football, basketball, handball, volleyball) to come in and play with inmates to break the monotony and increase motivation Enable occasional outings to practice sports externally, such as visiting a local table tennis or football club, to boost wellbeing
Spaces	Find a bigger training area that allows enough space to allow at least 10 people to work with weights Remove the sink in the boxing room (to prevent injuries) Expand the martial arts area Use part of the sports facility to add at least two treadmills Install a net barrier to prevent ball loss during racquetball play Add at least two showers (with running water) in the yard Reopen the gym/weight room in the module (instead of the phone booths, which don’t work either)	Paint a pickleball court, which could motivate some inmates to try the sport
Materials and equipment	Repair rowing machines Install wall bars Repair all non-functional exercise machines to ensure safe use Protect the weight training area so machines and equipment don’t get damaged Add one more punching bag Provide new weights and dumbbells and maintain existing ones Upholster and oil machines that need it Provide modules with a wider range of weights (currently jumps from 18 to 32 kg) Provide larger goalposts for futsal [a variant of football/soccer] Add volleyball posts and net Secure the pull-up bars properly (they move) Add a table tennis table	Provide basic sports materials (e.g., footballs, frontenis** balls or rackets, and weight training equipment) as many items are lacking or often get stuck/lost
Staff and support	Increase the number of sport technicians (mentioned above under activities – kept here for emphasis)	Increase the number of physical activity facilitators or volunteers, such as monitors who guide exercise sessions. Their presence improves motivation and participation
Limitations related to regulations	None reported by participants	None reported by participants
Schedule and restrictions in time allocation	Extend the hours of access to the sports facility to play futsal Organize inter-module sports matches	Resolve schedule conflicts between work duties and school classes, which currently prevent participation in physical activity at the sports facility Introduce flexible work shifts (e.g., alternating morning and afternoon) to allow inmates time for sports

Note: Strategies are presented in order of implementation difficulty, starting with those considered easier to implement and followed by those of higher complexity

^*^All strategies translated from Spanish/Catalan

^**^A racquet sport traditionally played on a *fronton* court, combining elements of tennis and squash

### Staff workshops

Two workshops were conducted with prison staff and the policymaker to review the inmate-generated strategies. Staff evaluated the feasibility of strategies within institutional, operational, and budgetary constraints. This process produced a refined list of strategies approved for implementation, including budget allocation for maintenance of sport equipment, twice-weekly instructor-led sessions targeting inactive inmates, and introducing of a second weekly sports facility time slot. Staff input allowed strategies to be adjusted for operational feasibility while retaining alignment with inmate-identified needs.

Combining qualitative insights from workshops and real-world observations from the Discovery Tool produced an integrated framework of environmental and institutional factors affecting movement. Each theme connects to strategy clusters, illustrating how physical spaces, materials, staff support, regulations, and schedules interact to influence opportunities for movement within the prison setting.

## Discussion

This study aimed to identify built environment strategies within a prison setting that could promote movement—specifically by increasing PA and reducing SB—through a participatory co-creation process using the Our Voice community (citizen) science method. The findings revealed several top-priority and low-difficulty strategies identified by incarcerated participants, including improvements in access to sports equipment, better maintenance of existing fitness facilities, creation of more functional spaces for diverse activities, a wider range of activities led by qualified sports facilitators, and flexible scheduling that accommodates activity participation. These findings underscore the potential of low-cost, context-sensitive environmental changes to significantly impact health behaviors, even in highly regulated institutional settings such as prisons.

Although the literature on built environment influences on PA in prison settings is scarce, these findings align with broader evidence from community-based studies showing that access to well-maintained, attractive, and safe physical spaces supports increased PA [30–32]. Features such as exercise equipment, open recreational areas, and multi-use courts have been consistently associated with higher PA levels in schools, neighbourhoods, and workplaces [32, 33]. Similarly, in our study, participants emphasized the need for better equipment and maintenance (e.g., weights, balls, and pull-up bars), protected and appropriately sized spaces for exercise, and access to organized activities, suggesting that environmental modifications can help overcome motivation barriers and increase engagement [34, 35].

Importantly, several of the proposed strategies also highlight the potential for enhancing social connection, which is increasingly recognized as a critical determinant of mental health and PA participation [36–38]. Activities such as team sports, group exercise sessions, and shared spaces for recreation can foster peer support, collective motivation, and a sense of belonging—factors that not only encourage sustained PA but also mitigate the social isolation and reduced quality of life often experienced in prison environments [38]. This is particularly relevant in correctional settings, where mental health challenges are prevalent, and opportunities for meaningful social interaction are limited [39–41]. By facilitating environments where individuals can support and inspire each other to be more active, these strategies may yield dual benefits for both physical and mental health outcomes [37, 40].

The successful implementation of the Our Voice community science method in this study further underscores the feasibility and value of participatory approaches in correctional settings. Despite often being excluded from decision-making processes that affect their daily lives, incarcerated individuals demonstrated a high level of engagement, insight, and motivation when given the tools to evaluate and co-design their environment. This participatory process not only generated actionable strategies but also fostered a sense of agency and ownership, which are known to be critical drivers of sustained health behavior change [42]. Engaging vulnerable populations such as incarcerated adults in community science processes contributes to more equitable health promotion and can improve trust and communication between institutional authorities and residents [43]. As more physically active and healthier populations also typically incur less health care costs overall [44], the potential economic benefits to society of such potentially low-cost environmental changes merit further investigation.

The inclusion of participants with varying PA levels ensured that the strategies reflected diverse needs and preferences, further strengthening the relevance and potential impact of the proposed interventions. While this study is context-specific—conducted within a single prison—it provides a scalable and adaptable framework that could be applied in other correctional facilities or similarly restrictive institutional environments [45]. The use of participatory tools to assess and modify the built environment can be integrated into broader health promotion strategies, especially in settings where traditional top-down approaches have had limited success.

### Limitations

As with many co-creation processes, the findings are context-specific, reflecting the unique conditions, resources, and social dynamics of a single correctional facility. This may limit the generalizability of specific strategies, though the co-creation and community science approach itself is adaptable and can be applied in other institutional settings [45].

A potential limitation is that participants may have withheld critical views due to perceived institutional risks. To address this, workshops were conducted separately from prison staff, and data were collected and analyzed anonymously to promote open dialogue and confidentiality. The study focused exclusively on incarcerated males, reflecting the prison’s demographic composition and institutional priorities. Future research should explore gender-specific needs, including female and youth populations.

Finally, the use of a mobile app–based data collection tool introduced constraints. Researcher-provided devices were used under supervised conditions due to security protocols, which may have limited spontaneous documentation of some environmental features. These factors should be considered when assessing the feasibility and transferability of digital participatory methods in other correctional contexts.

## Conclusions

This study demonstrates the feasibility and value of engaging incarcerated adults as community scientists to identify context-specific strategies for promoting PA and reducing SB. Through the Our Voice community science process, inmates, staff, and management collaboratively generated actionable interventions targeting both environmental and institutional factors within the prison setting. Strategies varied by activity level: active inmates prioritized autonomy, access to equipment, and facility use, while less active inmates emphasized structured support and motivational strategies.

Initial outcomes suggest that participatory approaches can foster staff recognition of inmate perspectives, strengthen institutional responsiveness, and support trust-building within correctional environments. Early indications of impact include plans to implement more flexible facility schedules, increase equipment availability, and enhance staffing in targeted units, contingent on measurable improvements in physical and emotional well-being.

The integrated framework developed from qualitative workshops and real-world observations illustrates how spaces, materials, staff support, regulations, and schedules interact to shape movement opportunities in prison. These findings provide a structured roadmap for designing participatory, health-promoting interventions and offer a scalable model for future research. Upcoming studies aim to evaluate the effectiveness of these strategies in randomized controlled designs and extend community science approaches to female and youth populations in correctional settings.

## Data Availability

The datasets used and/or analysed during the current study are available from the corresponding author on reasonable request.

## Abbreviations

COREQ: Consolidated Criteria for Reporting Qualitative Research
PA: Physical activity
SB: Sedentary behavior
